# Concealing Organic Neuromorphic Devices with Neuronal‐Inspired Supported Lipid Bilayers

**DOI:** 10.1002/advs.202305860

**Published:** 2024-05-03

**Authors:** Chiara Ausilio, Claudia Lubrano, Daniela Rana, Giovanni Maria Matrone, Ugo Bruno, Francesca Santoro

**Affiliations:** ^1^ Center for Advanced Biomaterials for HealthCare@CRIB Istituto Italiano di Tecnologia Naples 80125 Italy; ^2^ Dipartimento di Chimica Materiali e Produzione Industriale Università di Napoli Federico II Naples 80125 Italy; ^3^ Faculty of Electrical Engineering and Information Technology RWTH Aachen 52072 Aachen Germany; ^4^ Institute of Biological Information Processing – Bioelectronics IBI‐3 Forschungszentrum Juelich 52428 Juelich Germany; ^5^ Present address: Department of Biomedical Engineering Northwestern University Evanston IL 60208 USA

**Keywords:** neurohybrids, neuromorphic devices, organic electrochemical transistor, supported lipid bilayer

## Abstract

Neurohybrid systems have gained large attention for their potential as in vitro and in vivo platform to interrogate and modulate the activity of cells and tissue within nervous system. In this scenario organic neuromorphic devices have been engineered as bioelectronic platforms to resemble characteristic neuronal functions. However, aiming to a functional communication with neuronal cells, material synthesis, and surface engineering can yet be exploited for optimizing bio‐recognition processes at the neuromorphic‐neuronal hybrid interface. In this work, artificial neuronal‐inspired lipid bilayers have been assembled on an electrochemical neuromorphic organic device (ENODe) to resemble post‐synaptic structural and functional features of living synapses. Here, synaptic conditioning has been achieved by introducing two neurotransmitter‐mediated biochemical signals, to induce an irreversible change in the device conductance thus achieving Pavlovian associative learning. This new class of in vitro devices can be further exploited for assembling hybrid neuronal networks and potentially for in vivo integration within living neuronal tissues.

## Graphical abstract

Bio‐inspired supported lipid bilayer can conceal organic neuromorphic devices allowing for the seamless integration with biological tissue. Furthermore, the electrical and mechanical properties of the membranes can be tuned to regulate ionic communication and faradic charge transfer in organic neuromorphic transistor, regulating neurotransmitter‐mediated synaptic conditioning.

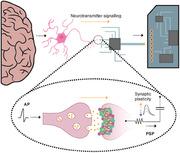

## 1 Introduction

In the last years, neuroelectronic devices have been engineered for in vitro and in vivo applications and optimized for a seamless integration of various cell types and tissue of the nervous system.^[^ ^1^ ^]^ In this context, resembling structural and functional features of neuronal cells into material design and device operation has posed major challenges.^[^ ^2^ ^]^ Here, the creation of neuronal‐like conductive materials has been exploited by patterning vertical structures to emulate dendritic spine as well as fabricating fiber‐based assembly, mirroring neurites and branching out architectures.^[^ ^3^ ^]^ Moreover, to recapitulate neuron‐neuron communication and adhesion processes mediated by the plasma membrane, artificial bilayers have been also used as suitable in vitro cell culture platforms for neuronal cells.^[^ ^4^, ^5^, ^6^, ^7^, ^8^ ^]^ Here, the lipid composition and charge can modulate the neuronal outgrowth and development inhibiting or supporting the neurites sprouting.

Recently supported lipid bilayers (SLBs), *i.e*., artificial biomembranes assembled on solid supports like metals or polymers,^[^ ^6^, ^9^, ^10^ ^]^ found extensive application in designing biomimetic platforms.^[^ ^1^ ^]^ Notably, such biomembranes can host native protein assemblies composing the cellular membrane by insertion or including cellular blebs to artificial lipids.^[^ ^11^, ^12^ ^]^

In addition, the possibility to assemble SLBs on organic mixed ionic/electronic conducting materials (OMIECs), enforces new frontiers in the field of biosensing to transduce signal from ionic sensitive systems, as proteins and ion channels.^[^ ^13^, ^14^ ^]^ In this context, the formation of artificial membranes on OMIECs can be facilitated by a microfluidic channel with certain geometrical requirements, to be able to induce lipidic vesicles rupture,^[^ ^9^, ^15^ ^]^ or additional “cushions”, like polyethylene glycol (PEG) to reduce proteins frictional coupling with the support underneath.^[^ ^11^, ^16^, ^17^, ^18^ ^]^

On the other hand, OMIECs have been recently integrated into chip‐based memristive devices like electrochemical neuromorphic organic devices (ENODes) that can resemble neuronal functions such as plasticity and learning capabilities^[^ ^2^, ^19^, ^20^ ^]^ and could be interfaced with diverse biological systems.^[^ ^21^, ^22^ ^]^ These three‐terminal devices based on organic electrochemical transistors (OECTs) also exhibited a response to individual or multiple neurotransmitters, leading to a non‐volatile conductance modulation, mirroring the long‐term memory conditioning observed in biological synapses.^[^ ^23^, ^24^, ^25^ ^]^

Recently, SLBs have been assembled on poly(3,4‐ethylenedioxythiophene):polystyrene sulfonate (PEDOT:PSS) based‐OECTs to develop biohybrid platform able to replicate brain functionalities, including short‐term and long‐term memory effects.^[^ ^23^ ^]^ Importantly, the incorporation of artificial biomembranes proved to improve the short‐term depression behavior and to resemble the brain's short‐term plasticity.^[^ ^23^ ^]^

However, such devices still hardly recapitulate the complexity of the synaptic architecture, particularly the presence and specific roles of pre‐ and post‐synaptic terminals.

In this work, a neurohybrid platform is proposed by assembling an artificial neuronal‐like biomembrane directly on an ENODe. Here, SLBs were exclusively confined to the post‐synaptic region of the artificial neuron which improved the conductance variation of the device. Furthermore, serotonin (5‐HT) and dopamine (DA) neurotransmitters have been introduced to achieve a non‐volatile conductance modulation and finally, Pavlovian associative learning was also demonstrated.

Such SLBs‐coupled OECTs, mimicking essential aspects of learning and memory processes in the brain, could offer exciting opportunities to develop artificial neuronal networks and advanced biohybrid systems, with potential application in neuroscience research.

## 2 Results and Discussion

ENODEs were fabricated as previously shown^[^ ^23^ ^]^ by using glass substrates with indium tin oxide (ITO) electrodes, where Poly(3,4‐ethylenedioxythiophene) (PEDOT:PSS) polymeric gate electrode (pre‐synaptic terminal) and channel were deposited through a selective dry etching procedure (Experimental Section). Then, an SLB reconstituting the composition of the biological neuronal plasma membrane^[^ ^26^ ^]^ has been assembled at the ENODe channel: here, the SLB‐PEDOT:PSS channel represented the post synaptic terminal of the hybrid synapse. Two types of SLBs were formed, one type starting from 1‐palmitoyl‐2‐oleoyl‐sn‐glycero‐3‐phosphocholine (POPC) lipid, the most frequently found component of biological membranes,^[^ ^27^ ^]^ and the other (namely brain‐SLB) assembled from POPC with the addition of cholesterol (chol) and sphingomyelin (SM) (1:1:1 in mixture), the major constituents of neuronal lipid rafts.^[^ ^28^, ^29^ ^]^ Notably, such biomembranes were formed by solvent assisted lipid bilayer (SALB) technique^[^ ^15^ ^]^ and exclusively confined on the polymeric channel by using a dedicated microfluidic module made of polydimethylsiloxane (PDMS).

**Figure 1:**
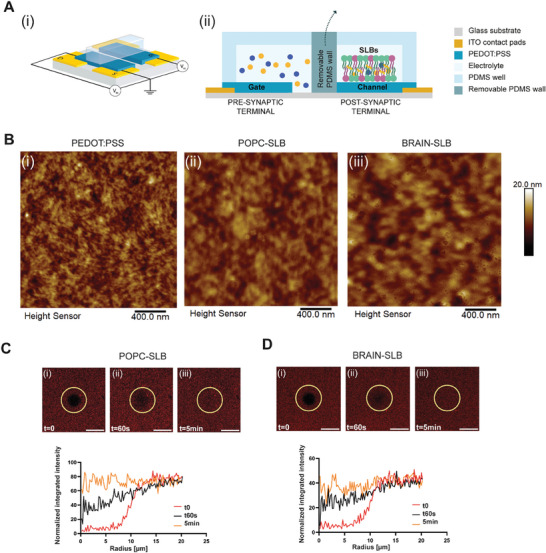
(A) Schematics of the device: i) 3D isometric projection and ii) side view. B) AFM images of bare PEDOT:PSS, POPC‐containing bilayer, and POPC‐chol‐SM‐ containing SLB (1:1:1 in mixture). C) FRAP results of POPC‐bilayer: fluorescence intensity recovery after photobleaching at t = 0 s, 60 s, and 5 min and the corresponding fluorescence intensity profiles. D) FRAP results of POPC‐chol‐SM (Brain) SLB: fluorescence intensity recovery after photobleaching at t = 0 s, 60 s, and 5 min and the corresponding fluorescence intensity profiles.

Following the formation of the membrane, the PDMS wall separating the device terminals was removed, allowing for the electrolyte to interface with both gate electrode and SLB‐covered channel (**Figure** **1**A; Figure S1, Supporting Information).

Here, the surfaces morphology and the local roughness of SLBs were investigated by means of atomic force microscopy (AFM, Figure 1B). As previously reported,^[^ ^23^ ^]^ the average roughness (R_a_) of bare PEDOT:PSS (1.45 ± 0.02 nm) is higher of the one characterizing the PEDOT:PSS/POPC‐ bilayer surface (1.37 ± 0.03 nm), confirming that the membrane completely covers the polymeric film (Table S1, Supporting Information). On the contrary, the interface offered by PEDOT:PSS/POPC‐chol‐SM‐ containing SLBs, was characterized by a higher surface roughness, compared to the one of PEDOT:PSS (1.62 ± 0.02 nm), possibly due to the formation of liquid ordered domains, containing sphingomyelin and cholesterol, protruding out of the bilayer surface.^[^ ^30^, ^31^, ^32^, ^33^, ^34^ ^]^

Then, the integrity and the fluidity of SLBs on the PEDOT:PSS channel were investigated by labelling the membrane with the fluorescent compound Texas Red 1,2‐dihexadecanoyl‐sn‐glycero‐3‐phosphoethanolamine, triethylammonium salt (Texas Red DHPE) lipid. Here, confocal microscopy revealed the homogeneity of the bilayer in the compartmentalized microfluidic module. Moreover, the diffusion coefficient of the SLBs was investigated by fluorescence recovery after photobleaching (FRAP). Here, both bilayers showed the complete recovery of the fluorescence within 5 min, further confirming their correct formation (Figure 1C,D). Notably, POPC‐bilayer showed the higher diffusion coefficient (1.93 ± 0.15 µm^2^ s^−1^) compared to brain‐SLBs (1.25 ± 0.04 µm^2^ s^−1^, Table S1, Supporting Information), indicating that the presence of cholesterol and sphingomyelin domains might hinder the movements of lipids within the membrane.^[^ ^35^ ^]^

Subsequently, the electrical properties of SLBs were investigated by means of electrochemical impedance spectroscopy (EIS), in a two‐electrodes configuration where the working electrode was connected to the polymeric channel and the reference electrode was a non‐polarizable Ag/AgCl electrode (Experimental Section).

**Figure 2:**
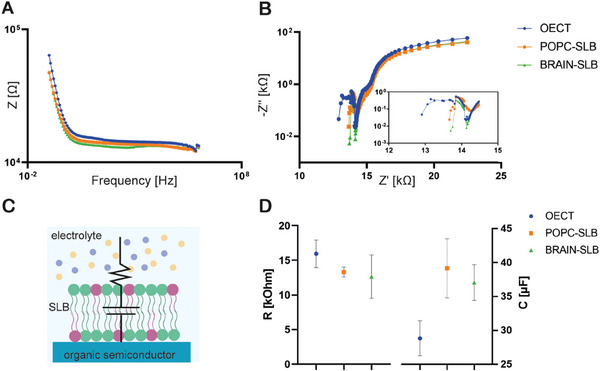
A) Bode and B) Nyquist plot of bare OECT, POPC, and POPC‐chol‐SM‐containing bilayers. C) Equivalent electrical circuit used for EIS fitting: the resistance described the electrolyte behavior and the capacitance accounted for the membrane. Capacitance and resistance numerical data were estimated by fitting EIS measurements with an electrical equivalent circuit composed by a resistance, accounting for the resistance of the electrolyte, and a capacitance, describing the capacitance of the electrode, connected in series D) Numerical values of the resistance and capacitance of bare OECT, POPC, and POPC‐chol‐SM‐containing bilayers. The presence of the bilayer did not induce a significant change in either parameters, without any significative differences between the two compositions.

Bode and Nyquist plots (**Figure** **2**A,B, respectively) depict a slight variation after the formation of the bilayer. This was further characterized by fitting with, an electrical equivalent circuit (RC) accounting for the resistance of the electrolyte and the capacitance of the PEDOT:PSS channel and the SLB altogether (Figure 2C). Diffusion‐like processes were neglected (*i.e*, no Warburg element).

While SLBs on a PEDOT:PSS electrodes are usually modelled with an additional RC circuit connected in series to the equivalent circuit representing the polymeric electrode,^[^ ^23^ ^]^ the formation of the confined bilayer on the ENODe did not lead to any significant variation of the equivalent electrical circuit components values compared to the bare PEDOT:PSS case (Figure 2D), suggesting possible diffusive processes and leakage at the interface between the bilayer and the polymer surface. This was also confirmed when a POPC SLB was formed covering the whole device (Figure S2A,B, Supporting Information), where confined SLB possibly hinders less the passage of ions from the electrolyte towards the PEDOT:PSS channel. Moreover, fitted parameters before and after the formation of the bilayer were comparable, in agreement with the hypothesis that the confined SLB did not induce any additional hindrance to the passage of ions, from the electrolyte to the channel. Interestingly, the brain‐SLB exhibits an higher capacitance than the POPC‐SLB, possibly due to diverse bilayers’ fluidity that influences the membrane dielectric constant and thus the, capacitive behavior.^[^ ^36^ ^]^ Here, the brain‐SLBs were characterized by the presence of heterogenous lipid rafts domains; such microdomains influence the fluidity of the bilayer and create nanoscale variation in the membrane structure, introducing local changes in the dielectric environment.^[^ ^37^ ^]^

Furthermore, the electrical properties of the SLB may modulate the doping/de‐doping process of the PEDOT:PSS and consequently the ENODe response^[^ ^23^ ^]^ which could be investigated through the device ionic circuit, i.e., a resistance (electrolyte along with other contributions like contact resistance) in series with a capacitance^[^ ^38^ ^]^ (volumetric capacitance of the channel) and its response to a single voltage pulse applied as gate bias.

**Figure 3:**
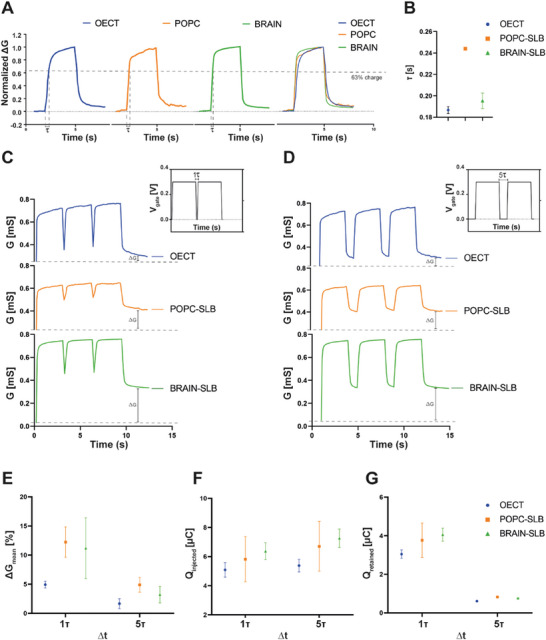
A) Calculation of the time constant τ of the ionic circuit between the gate and the channel of the OECT. The time constant corresponds to 63% of charge of the equivalent RC circuit modelling the OECT ionic circuit. The computation of τ was performed by applying a square voltage pulse at the gate electrode and monitoring the channel current to extract the time needed to charge the polymeric channel up to 63% of its maximum charged value. B) Mean values of the τ calculated before and after the formation of SLBs. C,D) The mean conductance variation elicited by gate voltage pulses with Δt equal to 1τ and 5τ with and without SLB. The graph highlighted how the presence of both bilayers increased the conductance modulation. E) Mean values of Δg computed before and after the formation of the SLBs and setting Δt equal to 1τ and 5τ. F,G) Percentage of charge injected/retained in the PEDOT:PSS channel was calculated with Δt equal to 1τ and 5τ. Mean values of charges injected/retained from the gate electrode to the channel was calculated after the application of a voltage pulse with Δt equal to 1τ and 5τ. The voltage bias applied at the gate terminal is 300 mV.

The time constant of the RC circuit, defined as τ (time to charge up to 63.2%, **Figure** **3**A), characterized the kinetics of the ion migration and accounted for several geometrical factors as well as surface interaction with SLBs.^[^ ^14^ ^]^

In fact, covering both gate and channel of the ENODe with a POPC SLB resulted in a significant increase of the time constant in comparison to the case without bilayer^[^ ^23^ ^]^ (Figure 3B; Figure S2C, Supporting Information). In contrast, when SLBs were confined on the ENODe channel, only a minor time constant variation can be observed (Figure 3B). In particular, the time constant of ENODes was increased after the formation of POPC‐SLB and was comparable to the one of BRAIN‐SLB. This suggested that the lower lipid mobility of BRAIN‐SLBs only slightly hinders gate‐channel ionic communication. Then, the neurohybrid synapse was characterized by applying an input bias at the gate electrode consisting of subsequent positive voltage pulses acting as a pre‐synaptic action potential‐like signal which would drive ions from the electrolyte into the ENODe channel causing a reversible modulation of its conductance.^[^ ^23^, ^38^ ^]^ Here, the interval between two consecutive input pulses (Δt) has been defined as multiple values of τ^[^ ^23^ ^]^ and the resulting conductance variation calculated before and after the application of the input signal represented the short‐term depression (STD) of the ENODe.^[^ ^23^, ^24^, ^39^ ^]^

Figure 3C,D depict the conductance variation of the ENODe with bare PEDOT:PSS channel and in presence of the confined SLBs considering Δt = τ and Δt = 5 τ, respectively.

In the first case, the presence and the confinement of the bilayer displayed higher conductance modulation compared to bare devices, without significative differences between the two lipid compositions (Figure 3E). This confirmed that the presence of the confined SLBs might promote the ion trapping, hindering the discharge of the PEDOT:PSS channel in contrast to the case where the SLB covered the whole device.^[^ ^23^ ^]^ Therefore, the presence of the confined brain‐SLBs would enhance the short‐term memory effect of the neurohybrid synapse.

To further corroborate this hypothesis, the number of charges injected by the gate and retained within the channel was quantified before and after the formation of SLBs (Figure 3F,G, respectively). As expected, the higher number of charges was measured when Δt was equal to τ, as, after the application of a voltage pulse, the channel was still partially de‐doped when applying consecutive voltage pulses, and therefore negative charged of PSS^−^ molecules could be progressively compensated by cations entering the bulk of the polymer. This result was obtained for both bare and SLBs‐coated ENODes. On the contrary, when Δt was equal to 5τ, the channel was almost completely doped and the number of ions retained in the polymeric channel did not depend on formation of the bilayer. These results suggested that the biomembrane, even while not increasing the resistive ionic pathway, enhanced the charge retention inside the channel.

Then, neurotransmitter‐mediated synaptic plasticity (long term depression, LTD) was recapitulated through an oxidative reaction of the DA and 5‐HT neurotransmitters at the gate terminal.^[^ ^23^ ^]^

As a result, given that the gate electrode was biased to a potential able to induce neurotransmitter oxidation, protons and electrons generated in the redox reaction would migrate inside the transistor channel, reducing the PEDOT:PSS, changing its conductance level in a non‐volatile manner, and restoring charge balance.^[^ ^23^ ^]^ As the channel conductance variation was the effect of PEDOT:PSS reduction, the reverse effect was induced by exposing the polymer to oxygen, by, for instance, introducing hydrogen peroxide as electrolyte solution.^[^ ^39^ ^]^

**Figure 4:**
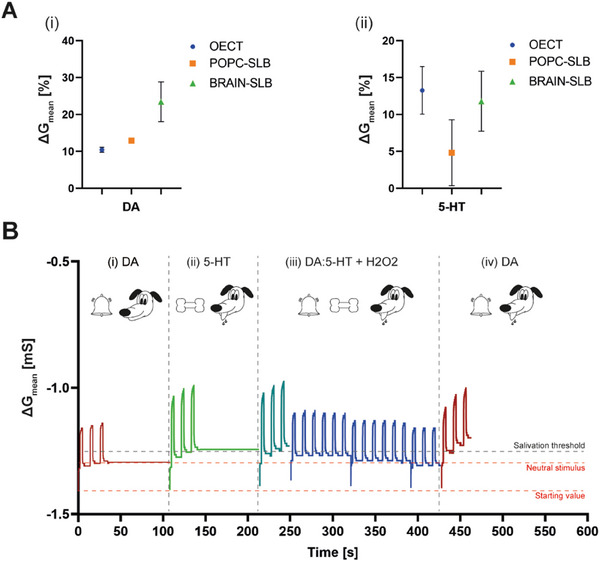
A,B) DA and 5‐HT‐mediated conductance modulation, respectively. The concentration employed for both neurotransmitters is 30 µM. C) Pavlovian associative learning experiment, recapitulated in a neuronal‐inspired SLB‐coated OECT.

The conductance variation in presence of 30 µM DA in the electrolyte before and after the formation of the SLBs onto the ENODe is shown in **Figure** **4**A‐i. Notably, the presence of the bilayer did not affect the neurotransmitter redox reaction, and the conductance modulation increased in presence of both POPC brain SLBs when compared to bare PEDOT:PSS.

Notably, while no significant differences could be observed among different SLBs compositions when oxidizing DA, 30 µM 5‐HT mediated conductance modulation of the POPC‐coated ENODe was lower than the brain‐SLB‐coated one (Figure 4A‐ii).

Such conductance modulation difference could be ascribed to the interaction between the fluidity of the bilayer and a strong fouling effect, typical of 5‐HT oxidation.^[^ ^40^, ^41^ ^]^ Previous studies reported that a continuous refresh of 5‐HT available species at the oxidation site drastically reduces the fouling effect,^[^ ^24^ ^]^ while here, the presence of the fluid SLB could prevent from a complete washing of the electrode surface in between consecutive measurements.

Finally, associative learning has been demonstrated by replicating the Pavlovian classical conditioning experiment (Figure 4B; Figure S3, Supporting Information) in which a dog was initially subjected to a neutral stimulus (bell ringing), without eliciting any response. Then, the subject undergoes an unconditioned stimulus (food) that evokes an unconditioned response (salivation). Finally, the two stimuli were delivered altogether. After some iterations, the dog associates the neutral stimulus with the unconditioned one. The associative learning is complete when the neutral stimulus can elicit the unconditioned response: the dog starts to salivate whenever the bell rings.^[^ ^42^ ^]^

Here, the device featuring the brain‐SLB was first exposed to the neutral stimulus (DA [30 µM]), while applying three consecutives voltage pulses at the gate terminal (Figure 4B‐i), defining the neutral stimulus response (minimum conductance level reached after the pulsing). Then a washing procedure was performed by introducing H_2_O_2_ in the electrolyte solution (Experimental Section, Figure S3, Supporting Information).

The unconditioned stimulus (5‐HT [30 µM]) was then supplied to the device, along with three consecutives voltage pulses (Figure 4B‐ii), defining the salivation threshold as the minimum conductance value reached upon the neurotransmitter‐mediated modulation. The unconditioned response was correctly evoked whenever the conductance reached or overcame this threshold. After an additional washing procedure (restoring the initial conductance level) the associative learning step was performed. Three voltage pulses were applied at the gate terminal, while using a DA:5‐HT binary solution as electrolyte (ratio of neurotransmitter 1:1, [30 µM:30 µM]) followed by a washing procedure (Figure 4B‐iii). After the combined stimuli, the associative learning was completed, by changing the baseline conductance value (Figure S3, Supporting Information). Indeed, by supplying one more neutral stimulus (DA [30 µM]), the conductance level of the neuromorphic device crossed the previously defined salivation threshold (Figure 4B‐iv).

Pavlovian learning experiments were carried out using both bare PEDOT:PSS and POPC‐coated ENODes (Figure S3, Supporting Information). Notably, associative learning could not be recapitulated in ENODes featuring POPC SLBs, because of the reduced 5‐HT‐mediated conductance variation. As a result, the neuronal‐inspired membrane excelled in the recapitulation of synaptic plasticity and brain‐inspired learning.

## 3 Conclusion

Bioelectronics devices represent a promising approach to probe both in vivo and in vitro systems. In this context, neuroelectronics devices were proven to be ideal candidates especially for bio‐interfacing applications. Nonetheless, the growing need to optimize cell‐chip coupling has led to the development of new classes of smart devices, able to resemble biological structural and functional features. Indeed, organic neuromorphic devices were shown to interface living tissue, while exhibiting computational features of neural communication. In this work neuronal‐inspired SLBs were exclusively confined to the artificial post‐synaptic terminal of an ENODe, enhancing its biomimetic capabilities by physically modulating ionic movement, thus allowing to control the mechanism of short‐term and long‐term plasticity. This latter mechanism was emulated exploiting DA and 5‐HT as biorelevant cues. Here the neuronal‐inspired composition emerged as best suited in applications at the border between biology and electronics, as it does not hamper electrochemical reactions that usually occurs in biological signaling, enabling the recapitulation of Pavlovian associative conditioning. These biohybrid devices, mimicking both functionalities and composition of neurons, may pave the way toward the realization of neuronal‐inspired technologies which are optimally integrated with living tissue, with potential applications in the field of neuroscience.

## 4 Experimental Section

### ENODe Fabrication

Glass substrates (25 × 25 mm^2^), with four indium tin oxide (ITO) squares (10 × 10 mm^2^) at each corner (Xinyan Technology Ltd., China) were used. PEDOT:PSS aqueous solution (Hereaus, Clevios PH1000, Germany) was mixed with 5 vol% ethylene glycol (Sigma‐Aldrich, USA), 1 vol% 3‐glycidyloxypropyl)trimethoxysilane (Sigma‐Aldrich, USA), and 0.02 vol% dodecylbenzene sulfonic acid (Sigma‐Aldrich, USA). The blended PEDOT:PSS solution was deposited on the glass substrate, previously treated with oxygen plasma (Tecno‐Service, Italy) for 2 min at 20 W. The spin coating was performed at 2000 rpm for 2 min, followed by thermal annealing at 140 °C on a hot plate, 1 h. Thus, the thickness of the spin coated film was ≈110 nm. Two symmetrical strips (7 × 17 mm^2^) representing the gate and the channel were obtained through oxygen plasma dry etching technique for 15 min, at 100 W by using physical masks made of poly(dimethylsiloxane) (PDMS, Sylgard 184) mixed in ratio 10:1 w/w with a cross‐linker and cured at 80 °C for 1 h. then. Then, the PEDOT:PSS was swelled by immersing the devices in milli‐Q water, for 1 h.

### Microfluidics Module and Removal Procedure

The two parts constituting the microfluidic module were made by means of negative polymethyl methacrylate (PMMA, Goodfellow, USA) molds, created using a micromilling machine (Minitech Machinery, USA). The actual module was realized using poly(dimethylsiloxane) (PDMS, Sylgard 184) mixed in ratio 10:1 w/w with a cross‐linker, cured at 120 °C for 1 h. First, an open (no roof) and rectangular‐shaped microfluidic channel (17 × 3.8 × 0.4 mm^3^) was created and mounted on the device through a two components glue (Picodent Twinsil, Germany). Then, a second PDMS layer, with a 1 × 3.8 × 0.4 mm^3^ protrusion (PDMS wall in the final module), was glued on top of the previous part, allowing to define two separated microfluidic chambers (7 × 3.8 × 0.4 mm^3^ and 9 × 3.8 × 0.4 mm^3^, respectively). In addition, the PDMS thin wall of the module was manually removed, to be subsequently glued on the device, in order to completely isolate the two microfluidic chambers. After the formation of the SLBs, the PDMS wall was gently detached from the substrate, connecting the two chambers.

### Electrical Impedance Spectroscopy (EIS) Measurements

Electrochemical impedance spectroscopy measurements were performed with an Autolab PGSTAT302N (Metrohm, Switzerland) potentiostat/galvanostat interfaced with a personal computer, equipped with the NOVA software. The ENODE channel acts as working electrode and a standard Ag/AgCl electrode (Redox.me, Sweden) works as both counter and reference electrode. Measurements were performed in a phosphate buffer solution (pH = 7.4) (Merck Life Science S.r.l., Italy). The sinusoidal input signal was set to 10 mV by scanning a range of frequencies from 1 Hz to 100 kHz. EIS data analysis and fittings were carried out through custom‐made Python scripts, exploiting SciPy and NumPy Python packages.

### Atomic Force Microscopy (AFM) Characterization of PEDOT:PSS Films

AFM measurements were performed with a Bruker Dimension Icon microscope (Bruker Corporation, USA) in ScanAsyst mode in hydrated conditions (TrisNaCl buffer solution). The ScanAsyst‐Fluid probe (Bruker Corporation, USA) with a spring constant of 0.7 N m−1, a tip radius of 5–20 nm, and resonance frequency of about 150 kHz was used. The scan rate was set at 2 Hz for 256 × 256 pixels images and the gain was optimized to reduce the noise. The root mean square (RMS) roughness (Rq) of PEDOT:PSS channel was determined using the provided analysis software Nanoscope Analysis 2.0 over 2 × 2 um areas. The images were plane fitted at order 0 and flattened at order 2.

### Liposomes Preparation

Lipids were dissolved in chloroform (Merck, Germany) at a desired concentration (10 mg ml^−1^). First, the chloroform was evaporated under a nitrogen stream and then in a desiccator under vacuum for 2 hours to remove any trace of solvent. The lipid film was rehydrated with a mixture of 70% v/v of water a 30% v/v of isopropanol to obtain a final concentration of 5 mg ml^−1^. The suspension was gently vortexed and sonicated on ice for 25 min. Then, the lipidic solution was extruded 15 times through a polycarbonate membrane with a pore size of 0.1 µm (Merck, Germany) by using a mini extruder (Sigma‐Aldrich, USA). The obtained lipid vesicle solution was stored at 4 °C. Two different lipids mixtures were investigated: one consisted of 100 mol% of 2‐Oleoyl‐1‐palmitoyl‐sn‐glycero‐3‐phosphocholine (POPC) (Avanti Polar Lipids, USA) and the other composed by POPC, cholesterol (Avanti Polar Lipids, USA) and brain sphingomyelin (Avanti Polar Lipids, USA) at a molar ratio of 1:1:1. 0.5% (mol mol^−1^) of Texas Red 1,2‐dihexadecanoylsn‐glycero‐3‐phosphoethanolamine triethylammonium (Thermo Fisher, USA) salt was incorporated in both lipid bilayers as a fluorescent probe.

### Supported Lipid Bilayer (SLB) Formation

The solvent assisted lipid bilayer (SALB) method was used to assemble lipid bilayers into a microfluidic channel.^[^ ^15^ ^]^ The latter, made of polydimethylsiloxane, mixed in ratio 10:1 w/w with its cross‐linker, cured at 80 °C for 1 h (PDMS, Sylgard 184), was used to selectively form the bilayer on the polymeric channel, owing the solvent exchange procedure. Briefly, the microfluidic module was composed of two unconnected compartments: the first one was incubated with lipid vesicles while the second one was filled with electrolyte. The two chambers were subsequently connected owing to a removable PDMS wall which allows the communication between the channel and the gate electrode. Prior to the SLB formation, the ENODe was treated with oxygen plasma (Diener electronic, Germany) for 2 min at a pressure of 1 mbar with power of 20 W. The liposome solution was diluted with a mixture of isopropanol and water (3:7 v/v) to a final concentration of 0.5 mg/ml and incubated for 30 min inside the first compartment of the microfluidic channel. The solvent was removed with a buffer solution (10 × 10^−3 ^M Tris, 100 × 10^−3 ^M NaCl, pH 7.5) with a flow rate of 50 µl min^−1^ for 2 h.

### Atomic Force Microscopy (AFM) Characterization of SLBs

AFM measurements were carried out on Bruker Dimension Icon microscope (Bruker Corporation, USA) in ScanAsyst mode. The scanning procedure was performed in hydrated condition (TrisNaCl buffer solution) by using the ScanAsyst‐Fluid probe (Bruker Corporation, USA), with a spring constant of 0.7 N m^−1^, a tip radius of 5–20 nm, and resonance frequency of 150 KHz. The scan rate was set at 1.5 Hz for 256 × 256 pixels images and the gain was optimized to reduce the noise. The images were processes by using the Nanoscope analysis 2.0 software and the average roughness (R_a_) was calculated over 2 µm x 2 µm areas.

### Fluorescence Recovery After Photobleaching (FRAP) Characterization

FRAP measurements were carried out with a TCS SP5 gated with stimulated emission depletion (STED) (Leica, Germany). The images were acquired by using a 25x water immersion objective with scanning speed of 1000 Hz. A circular spot with a diameter of 20 µm was bleached using a 114 mW 592 nm laser beam for 1.3 s and monitored for 5 min. The fluorescence intensity was normalized to a reference spot in each image and fit with a Bessel function.^[^ ^43^ ^]^ The fluorescence intensity profiles along the radius of the bleached spot were calculated at three different time points (0 s, 60 s, and 5 min) by using the ImageJ software (NIH, USA) through the Radial Profile Angle tool. The diffusion coefficient was calculated through the following formula:

(1) $$D=\omega^{2}/\left(4\tau_{\left(1/2\right)}\right)$$

with ω radius of the bleached area and τ1/2 the time required to achieve half of the maximum recovery intensity.

### Electrical Measurements

The electrical characterization was performed with the simultaneous use of two independent source measure units (SMUs) by using a commercial setup (Arkeo, Cicci Research, Italy). Pulsed measurements were performed by keeping the drain voltage equal to −0.2 V and by applying square voltage pulses on the gate electrode between 0 and 0.3 V. Each measurement consisted of three pulses with pulse width of 3 s and delay between pulses of 9 s. Custom made MATLAB scripts were used to process the time domain data.

### Time Constant Analysis

The time constants were computed by applying a square voltage pulse at the gate terminal, while monitoring the channel current. The numerical values were extracted as the time needed to reach the 63.2% of the minimum current value, starting from the application of the pulse.

### Neurotransmitters’ Solution Preparation

The solutions containing the individual neurotransmitter were freshly prepared by mixing dopamine hydrochloride (98%, Sigma‐Aldrich, USA) and serotonin hydrochloride (98%, Sigma‐Aldrich, USA) with TrisNaCl buffer solution at the concentrations of both DA and 5‐HT (30 µM). The binary mixture comprising both neurotransmitters was obtained by combining the solutions of the single neurotransmitters, keeping the concentrations equal to 30 µM.

### Neurotransmitters Measurements

Both DA and 5‐HT solutions were inserted in the microfluidic channel, after the removal of the PDMS wall separating the gate and the channel. Three pulses were applied at the gate terminal. In order to match the oxidation potential of each neurotransmitter,^[^ ^44^ ^]^ the amplitude of the pulses was set to 0.3 V in case of DA, and to 0.4 V in case of 5‐HT. Three consecutive measurements were performed by performing three washes with TrisNaCl buffer between measurements. The channel conductance variation was calculated as the ratio between the channel current and the applied voltage, and then averaged for each device. The measurements were performed for three independent devices (N = 3).

### Pavlov Experiment

The Pavlov conditioning measurements were performed following two different procedures. The first one was the learning procedure, in which a neurotransmitter solution (DA, 5‐HT or DA:5‐HT) was used as electrolyte. In this case 3 square pulses were applied at the gate terminal with amplitude 0.3 V (DA) or 0.4 V (5‐HT and DA:5‐HT). In the second procedure, defined as washing, H_2_O_2_ was present in the electrolyte solution, to oxidize the polymeric channel, reversing the neurotransmitter‐induced polymer reduction. Six voltage pulses of 0.3 V were applied at the gate terminal. Pulse width was equal to 3 s, while the delay between pulses was set to 9 s, in all cases (DA, 5‐HT, DA:5‐HT and H_2_O_2_).

## Conflict of Interest

The authors declare no conflict of interest.

## Author Contributions

C.A. and U.B. equally contributed to this work. C.A. and U.B. contributed equally to the experimental design, data acquisition, analysis. G.M. designed the associative learning experiment and contributed to data acquisition. D.R. and C.L. contributed to data acquisition. F.S. contributed to conceiving, funding acquisition, supervision, data analysis. All authors contributed to manuscript writing and revision.

## Supporting information

Supporting Information

## Data Availability

The data that support the findings of this study are available from the corresponding author upon reasonable request.
