# Supplementary material for: Concealing Organic Neuromorphic Devices with Neuronal‐Inspired Supported Lipid Bilayers
Source: Adv Sci (Weinh). 2024 May 3;11(27):2305860. doi: 10.1002/advs.202305860 (PMC11251551; doi:10.1002/advs.202305860)

ADVANCED SCIENCE
Open Access

Supporting Information

for *Adv. Sci.*, DOI 10.1002/advs.202305860

Concealing Organic Neuromorphic Devices with Neuronal-Inspired Supported Lipid Bilayers

*Chiara Ausilio, Claudia Lubrano, Daniela Rana, Giovanni Maria Matrone, Ugo Bruno and Francesca Santoro**

Supporting Information

Table S1. Roughness parameters of the OECT-channel, POPC-containing SLB and POPC-chol-SM-SLBs (BRAIN-SLB), both formed on the OECT-channel. Diffusivity parameters of POPC-containing SLBs and POPC-chol-SM-containing-SLBs.

	**Roughness R_q [nm]**	**Diffusion Coefficient [$\mu m^2/s$]**
**OECT-channel**	1.45 ± 0.02 nm	-
**POPC-SLB**	1.37 ± 0.03 nm	1.93 ± 0.15 $\mu m^2/s$
**BRAIN-SLB**	1.62 ± 0.02 nm	1.25 ± 0.04 $\mu m^2/s$

Figure S1. Microfluidic module assembly and SLB formation procedure. (**i**) The two parts of the microfluidic module were mounted on the ENODe. (**ii**) Two chambers were thus defined: SLB chamber, in which the SLB would be formed, and a second chamber, isolated from the first one. (**iii**) The PDMS wall was removed. (**iv**) The PDMS wall was glued on the ENODe, to ensure the isolation of the two chambers. (**v**) SLB was formed in the SLB chamber. (**vi**) The PDMS wall was removed again, connecting the two chambers.

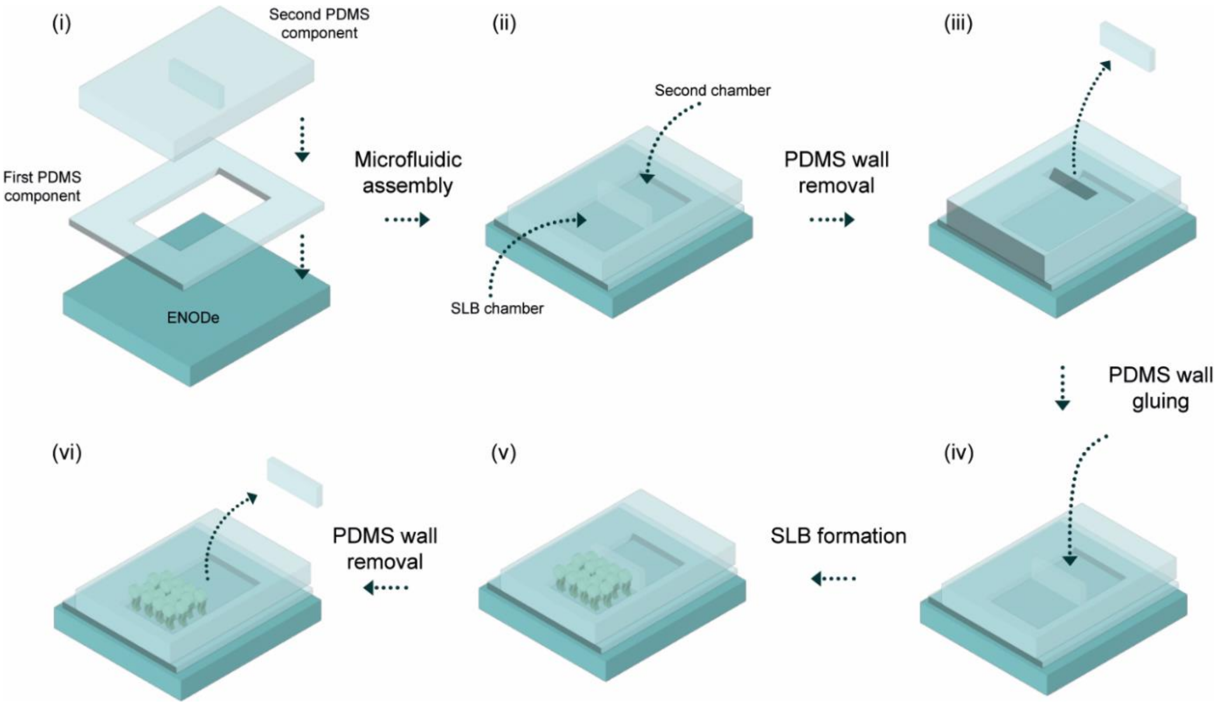

Figure S2. Numerical values of the (**A**) resistance and (**B**) capacitance of bare ENODe, both POPC and brain SLBs confined to the channel (one side -OS- configuration) and POPC-SLBs assembled along the entire microfluidic channel. The formation of the bilayer covering both the channel and the gate terminals (full configuration) induced significant change in the equivalent circuit parameters, extracted by fitting EIS measurements. (**C**) Mean values of the τ calculated before and after the formation of SLBs in both OS and full configurations.

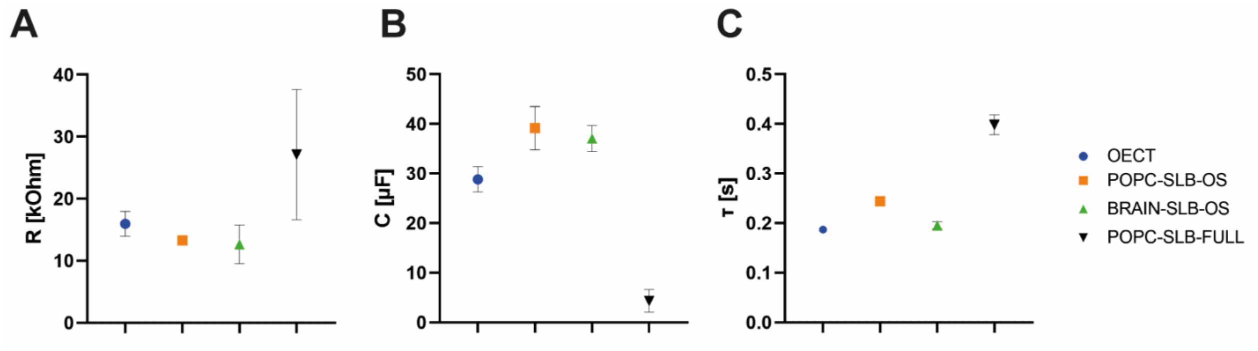

Figure S3. Pavlovian learning was recapitulated using (**A**) bare ENODEs and (**B**) POPC-coated OECT in one-side configuration.

Associative learning is achieved by applying the combined DA:5-HT stimulation. Here, given the increasing amount of oxidized species, the conductance variation is not reversed after the washing procedure, leading to non-volatile conditioning. Thus, it was possible to recapitulate

pavlovian associative learning using the bare OECT, while the POPC-coated transistor did not feature a strong 5-HT mediated conductance modulation. As a result, the response to the neutral stimulus and the salivation threshold could not be distinguished, preventing the correct recapitulation of the associative learning.

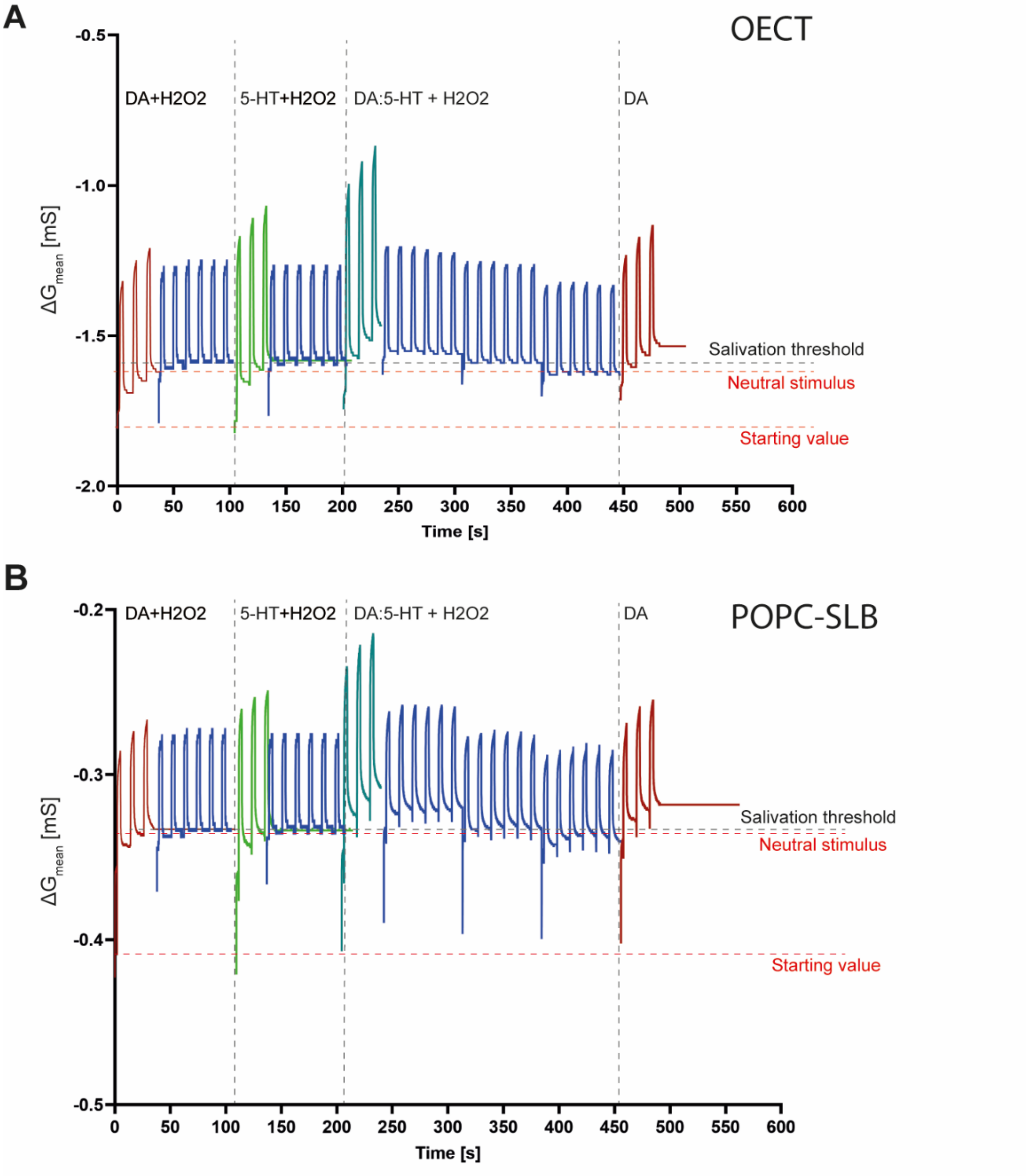

Supplement: Supplementary file 1 — Supporting Information [file ADVS-11-2305860-s001.pdf]
